# Co-culture of *Vel1*-overexpressed *Trichoderma asperellum* and *Bacillus amyloliquefaciens*: An eco-friendly strategy to hydrolyze the lignocellulose biomass in soil to enrich the soil fertility, plant growth and disease resistance

**DOI:** 10.1186/s12934-021-01540-3

**Published:** 2021-03-02

**Authors:** Valliappan Karuppiah, Lu Zhixiang, Hongyi Liu, Murugappan Vallikkannu, Jie Chen

**Affiliations:** 1 School of Agriculture and Biology, Shanghai Jiao Tong University, 800, Dongchuan Road, Minhang, Shanghai, 200240 PR China; 2 The State Key Laboratory of Microbial Metabolism, Shanghai Jiao Tong University, Shanghai, PR China

**Keywords:** Co-cultivation, *T. asperellum*, *B. amyloliquefaciens*, *Vel1*, Cellulase, Lignocellulose degradation

## Abstract

**Background:**

Retention of agricultural bio-mass residues without proper treatment could affect the subsequent plant growth. In the present investigation, the co-cultivation of genetically engineered *T. asperellum* and *B.* *amyloliquefaciens* has been employed for multiple benefits including the enrichment of lignocellulose biodegradation, plant growth, defense potential and disease resistance.

**Results:**

The *Vel1* gene predominantly regulates the secondary metabolites, sexual and asexual development as well as cellulases and polysaccharide hydrolases productions. Overexpression mutant of the *Trichoderma asperellum Vel1* locus (TA OE-*Vel1*) enhanced the activity of FPAase, CMCase, PNPCase, PNPGase, xylanase I, and xylanase II through the regulation of transcription regulating factors and the activation of cellulase and xylanase encoding genes. Further, these genes were induced upon co*-*cultivation with *Bacillus amyloliquefaciens* (BA)*.* The co-culture of TA OE-*Vel1* + BA produced the best composition of enzymes and the highest biomass hydrolysis yield of 89.56 ± 0.61%. The co-culture of TA OE-*Vel1* + BA increased the corn stover degradation by the secretion of cellulolytic enzymes and maintained the C/N ratio of the corn stover amended soil. Moreover, the TA OE-*Vel1* + BA increased the maize plant growth, expression of defense gene and disease resistance against *Fusarium verticillioides* and *Cohilohorus herostrophus*.

**Conclusion:**

The co-cultivation of genetically engineered T. *asperellum* and *B.* *amyloliquefaciens* could be utilized as a profound and meaningful technique for the retention of agro residues and subsequent plant growth.

## Background

Retention of agricultural bio-mass residues after harvest is an ideal strategy to improve sustainable agriculture [1]. Lately, onsite bio-degradation of crop residue has followed to maintain the soil fertility and to decrease the argumentative effects of residual burning in the agricultural field. Nonetheless, a few investigations showed that the retention of agricultural bio-mass residues affect soil properties and crop yields [2]. For instance, inadequate biomass degradation influences planting and seedling development, which augment the plant pests and pathogens [3, 4]. A promising solution for this issue is to inoculate the lignocellulolytic biomass degrading microbes into the soil. However, only a few studies have been focused on the onsite degradation of lignocellulolytic biomass using the microbes. Hence, it is the proper time to develop a technology for onsite biomass degradation, plant growth promotion and disease control.

*Trichoderma asperellum* has been considered as a beneficial fungus for plant growth, disease control, and the production of lignocellulolytic degrading enzymes [5]. Genetic engineering of the genes required for the regulation of lignocellulolytic enzyme synthesis of *Trichoderma* could provide an opportunity to improve both biomass degradation and plant growth. The expression of genes involved in lignocellulose degradation has been regulated by the co-ordination of numerous transcription factors [6]. Among them, *Vel1* positively regulates the cellulase production [7]. Karimi Aghcheh et al. [8] studied that the knockout of *Vel1* entirely declines the production and expression of cellulases related genes. In addition, the *Vel1* gene also regulate the morphogenesis, secondary metabolites and mycoparasitism of the *Trichoderma* [9].

Besides, co-cultivation technology is an advantage to stimulate the synergistic expression of metabolic pathways of two microbes [10]. Through co-cultivation, microbes develop different mechanisms to use substrates either by symbiotic or antagonistic interactions. The co-cultivation activates the silent genes and thereby induce the enzyme production. Substantial improvements have been made on the co-cultivation technology by co-cultivating the genetically engineered microbes to increase the production [11]. This methodology widens the prospects for the biosynthesis of complex proteins to utilize the natural substrates such as lignocellulolytic biomass. In our previous study, we proved that the co-cultivation of *B. amyloliquefaciens* 1841 and *T. asperellum* GDFS1009 activated several genes and induced the production of secondary metabolites and enzymes, including cellulase [12, 13]. *B. amyloliquefaciens* used in the co-cultivation is a plant growth promoting rhizobacteria [12–14]. In our previous study, we observed that the role of *T. atroviride Vel1* was enhanced by the *B. amyloliquefaciens* in the co-culture [9]*.* In light of the above findings, it has been anticipated that the application of the co-culture containing *Vel1* overexpression *Trichoderma asperellum* and *B. amyloliquefaciens* into the soil could enhance the genetic regulation on the cellulase and hemicellulase production and improve the bio-degradation of lignocellulolytic biomass in soil and subsequently improve plant growth and disease resistance of the plants grown in the same soil.

To prove our hypothesis, we developed the co-cultivation of *Vel1* over expressed mutant *T. asperellum* GDFS1009 and *B. amyloliquefaciens* 1841 (TA OE-*Vel1* + BA) to improve the cellulase production using the combination of genetic engineering and co-cultivation technology. We showed that the crude enzyme produced by TA OE-*Vel1* + BA enhanced the hydrolysis of corn stover biomass than the axenic culture. We further demonstrate that the co-culture of *T. asperellum* OE *Vel1* mutant and *B. amyloliquefaciens* enhanced the *in-vivo* lignocellulolytic degradation, plant growth, defense potential and disease control.

## Results

### Increased cellulase production by genetically engineering of *T. asperellum Vel1* gene

**Fig. 1:**
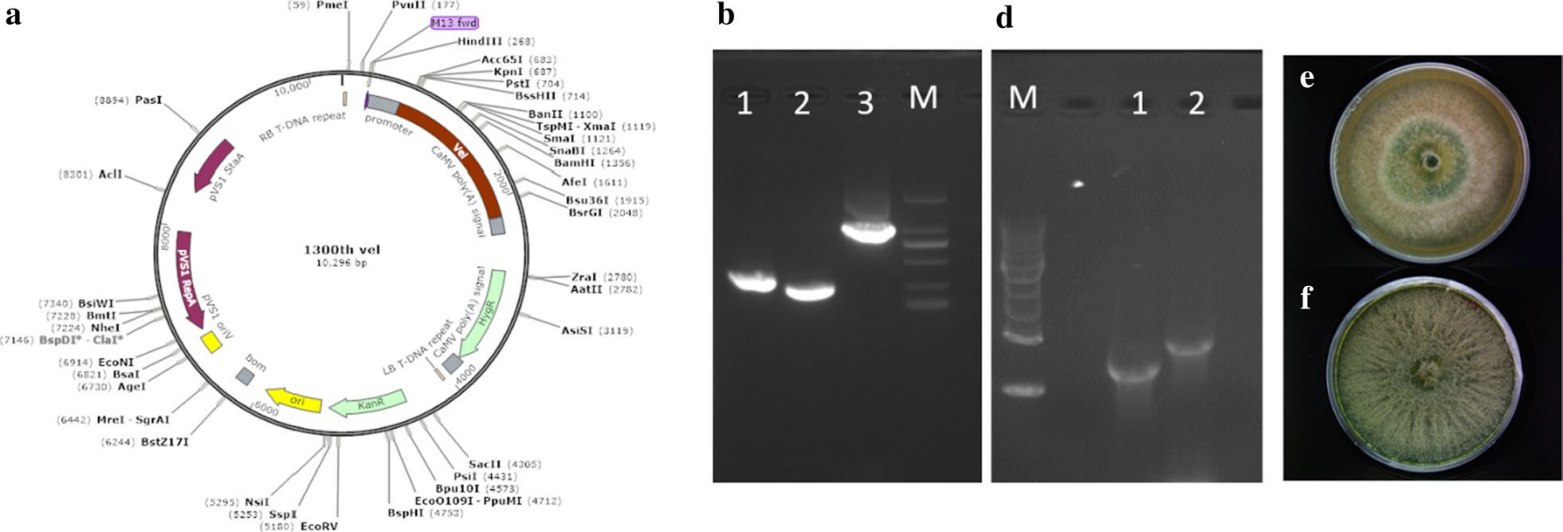
Overexpression of *Vel1* gene in *T. asperellum* GDFS1009. **a** Schematic diagram of the constructed plasmid pCAMBIA1300 -*Vel1*. **b** PCR amplification of TrpC promoter, *TrpC* terminator and *Vel1* ORF (1: *TrpC* promoter; 2: *TrpC* terminator; 3: *Vel1* ORF; M DS 2000 marker) **c** PCR results for transformant identification (M: 1 KB marker; 1: *Vel1* ORF; 2: over expression cassette containing *TrpC* promoter, *Vel1* ORF and *TrpC* terminator). **d** The phenotypes of Wild type (TA) and** e** phenotype of *Vel1* gene overexpress transformants grown on PDA plates

The strong promoter *TrpC* was used to improve the expression of *Vel1*. The over expression cassette containing *TrpC* promoter, *Vel1* ORF and *TrpC* terminator was cloned into pCAMBIA1300 and transferred to *T. asperellum* using *A. tumefaciens*-mediated transformation (ATMT). The recombinant vector pCAMBIA1300 *Vel1* OE and transformants are shown in Fig. 1. In total, 126 T*. asperellum* recombinant strains were obtained through the ATMT transformation. Among them, 5 recombinant strains showed higher filter paper activity (FPAse) than the wild type strain. The growth of *T. asperellum* recombinants on the cellulose containing medium was displayed in Additional file 1: Table S1. The fastest growing *T. asperellum* recombinants were selected among 5 transformants with the maximum cellulase activities.

### Influence of cellulase production by different samples

**Fig. 2:**
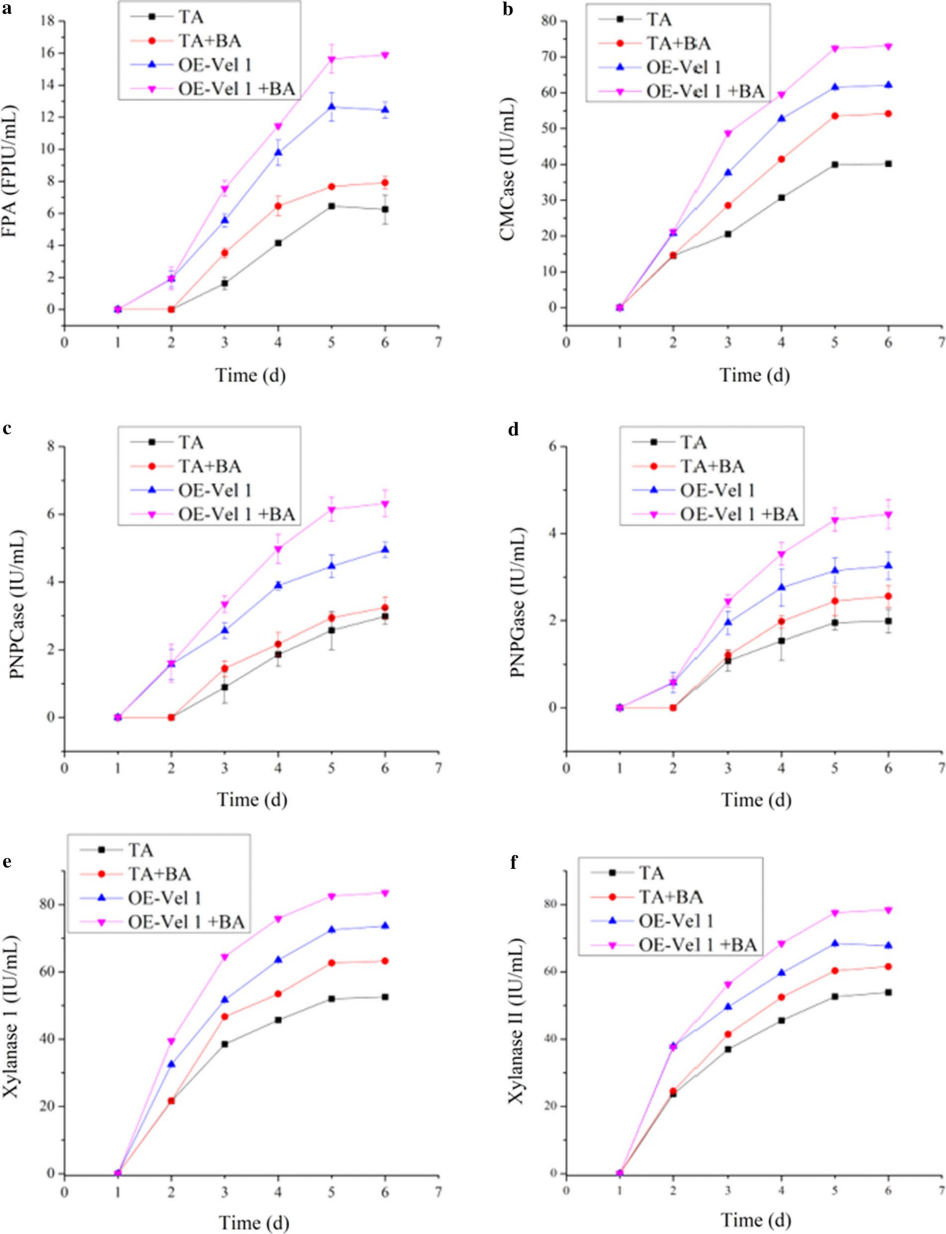
Comparison of cellulase production by different approaches.**a** FPase, **b** CMCase, **c** pNPcase, **d** pNPGase, **e** xylanase I, and **f** xylanase II. Values are the average of biological triplicates. Error bars represent the standard error

The four samples (Additional file 1: Fig. S1) such as the axenic culture of *T. asperellum*, co- culture of *T. asperellum* and *B. amyloliquefaciens*, the axenic culture of the TA OE-*Vel1*, and the co-culture of TA OE-*Vel1* and *B. amyloliquefaciens* were analyzed to know the best sample to produce lignocellulolytic enzyme for the degradation of lignocellulolytic biomass. The genetically engineered *T. asperellum* (TA OE-*Vel1*) showed higher activities of filter paper activity (FPAase), carboxymethyl cellulase (CMCase), 4-nitrophenylcellobiosidase (PNPCase), p-nitrophenyl-β-D-glucopyranoside (PNPGase), xylanase I and xylanase II (Fig. 2) compare to the *T. asperellum*, yielding values of 12.45 ± 0.05 FPIU, 62.14 ± 0.34, 4.95 ± 0.23, 3.26 ± 0.32, 73.67 ± 0.37, and 67.8 ± 0.36 IU/mL after 6 days of fermentation, respectively. On the other hand, the axenic culture of *B. amyloliquefaciens* failed to produce all of these activities. This revealed that the *Vel1* gene improved the activity of enzymes related to cellulose and hemicellulose hydrolysis. In-addition, *B. amyloliquefaciens* also induced the expression of lignocellulolytic enzymes [12]. The enzyme activities including FPAase, CMCase, PNPCase, PNPGase, xylanase I and xylanase II were enriched by the TA + BA compared to the TA (Fig. 2). The enzyme production might be owing to the different substrates. After 6 days of fermentation, the FPAase, CMCase, PNPCase, PNPGase, xylanase I and xylanase II activity of the co-culture of TA OE-*Vel1* and *B. amyloliquefaciens* were 7.92 ± 0.04 FPIU, 54.16 ± 0.46, 3.24 ± 0.32, 2.56 ± 0.25, 63.23 ± 0.37, and 61.57 ± 0.43 IU/mL respectively. It was identified that the co-cultivation of *T. asperellum* and *B. amyloliquefaciens* was a fantastic combination to obtain the higher activity of FPAase, CMCase, PNPCase, PNPGase, xylanase I and xylanase II activity. For the first time, in this investigation, the co-culture of the genetically engineered *T. asperellum* and *B. amyloliquefaciens* was attempted to synthesize the highest enzyme production by linking the recombination technology and co-cultivation. As shown in Fig. 2, the FPAase, CMCase, PNPCase, PNPGase, xylanase I and xylanase II activity of the co-culture of TA OE-*Vel1* and *B. amyloliquefaciens* were 15.91 ± 0.14 FPIU, 73.04 ± 0.16, 6.32 ± 0.39, 4.45 ± 0.32, 83.56 ± 0.43, and 78.45 ± 0.38 IU/mL respectively. These enzyme activities were considerably increased 1.1–1.3 fold than the TA OE-*Vel1.* Also, the enzyme activities were increased than the co-culture of *T. asperellum* and *B. amyloliquefaciens*. The results showed that this sample upgraded the synthesis of cellulase. Further, the results recommend that this kind of modified co-cultivation is more valuable than that of recombination technology and co-cultivation.

### Influence of the transcription regulating genes, cellulase and xylanase encoding gene expression by different samples

**Fig. 3:**
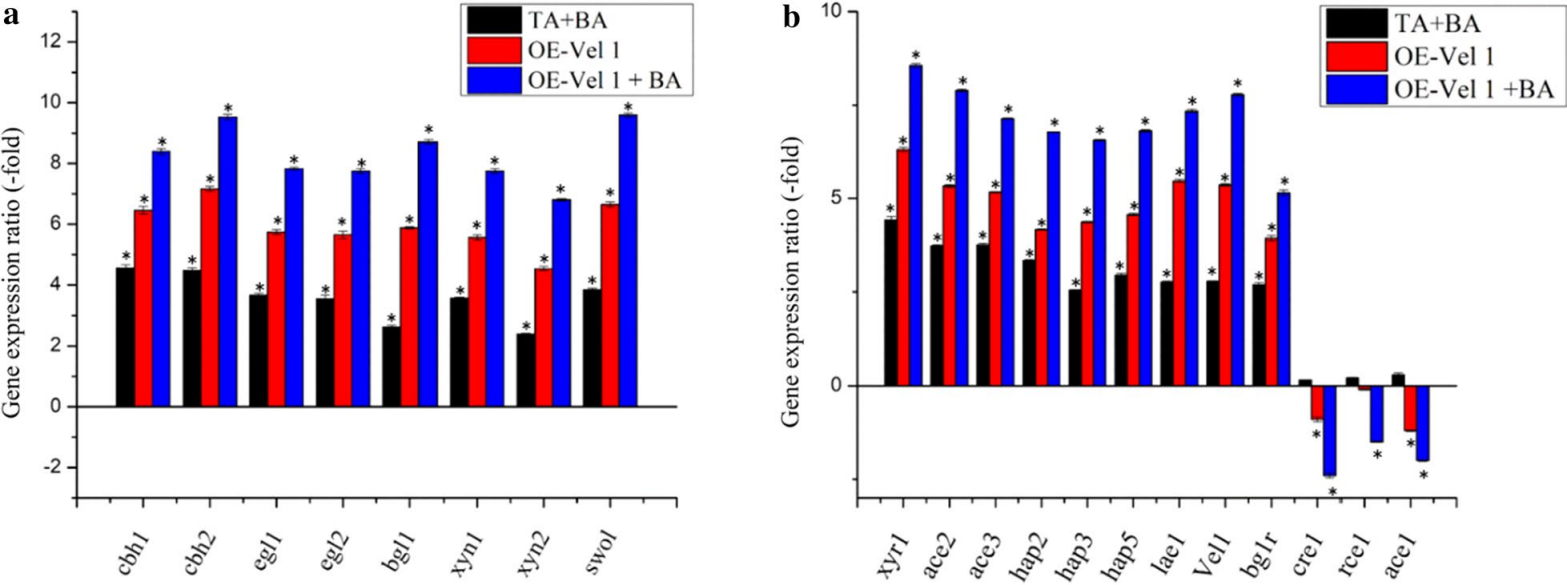
Transcriptional changes of the genes coding cellobiohydrolases, endoglucanases, β-glucosidase, xylanase, accessory proteins and transcription factors of different approaches. **a** expression of *cbh1*, *cbh2*, *egl1*, *egl2*, *bgl1*, *xyn1 xyn2* and *swo1*, **b** expression of transcription factors including *XYR1*, *ACE II*, *ACE III*, *BglR*, *Hap2/3/5*, *ACEI*, *RCE1*, and *CRE1*. Fold changes in TA + BA, TA OE-*Vel1* and TA OE-*Vel1* + BA were relatively compared to the wild type axenic culture (TA) at 72 h. Values are the average of biological triplicates. Error bars represent the standard error. Asterisks refer significant differences from monoculture of wild type strain (TA) (*p < 0.05, Student’s t test)

The expression pattern of *cbh1*, *cbh2*, *egl1*, *egl2*, *bgl1*, *xyn1* and *xyn2* were compared to know the regulatory level of cellobiohydrolases, endoglucanases, β-glucosidase and xylanase under different approaches. The expressions of these genes were strongly upregulated in the order of TA OE-*Vel1* + BA > TA OE-*Vel1* > TA + BA relative to the axenic culture of *T. asperellum* (Fig. 3). The expression level of the major cellulase gene, including cellobiohydrolases (*cbh1* and *cbh2*) endoglucanases (*egl1* and *egl2*), and β-glucosidase (*bgl1*) were similar to production of cellulolytic enzymes (Fig. 3a). The cellulase and xylanase encoding genes were coordinated by the group of transcription factors (TFs), including both inducer and inhibitors. The expression of cellulase regulatory genes by the *Vel1* has been explored by studying the expression of nine positive regulators and three repressor genes. The stimulation of cellulase was initially verified by the transcription analysis of *xyr1*, *ace II*, and *ace III*, which are the most important inducers of cellulase and xylanase production [15–17]. As shown in Fig. 3b, the relative quantification of the *xyr1*, *ace II,* and *ace III* gene were upregulated by the over-expression of the *Vel1* gene. The expression of *xyr1*, *ace II* and *ace III* were increased to 8.56, 7.98 and 7.14 fold, respectively in the TA OE-*Vel1* + BA then the axenic culture of *T. asperellum.* Meanwhile, the relative transcription folds of these genes were only 4.4, 3.7 and 3.7 in TA + BA. In addition, the transcription factors *BglR* and *Hap2/3/5* complex also positively regulated the cellulase and xylanase. The transcription level of the *BglR* and the *Hap2/3/5* complex was also upregulated in co-ordination with other genes. Among the negatively transcription regulating factors, *cre-1* is the carbon catabolite repressor, which completely inhibits the expression of the cellulase and xylanase genes [18]. Relatively, *ace1* inhibits the C2H2 zinc finger and negatively regulate the genes encoding cellulase and xylanase. Also, the *rce1* is the negative regulator by provoking Xyr1 [19]. To detect the influence of *ace I*, *rce 1*, and *cre 1*, the expression level of these genes was quantified. The results showed that these genes were downregulated with the overexpression of the *Vel1* gene and TA OE-*Vel1* + BA. The downregulation of *ace I*, *rce 1*, and *cre 1* might be involved in the upregulation of *cbh1*, *cbh2*, *egl1*, *egl2*, and *bgl1* through the overexpression of *Vel1* gene [20].

### Hydrolysis of cellulosic biomass by the differently sourced cellulases

**Fig. 4:**
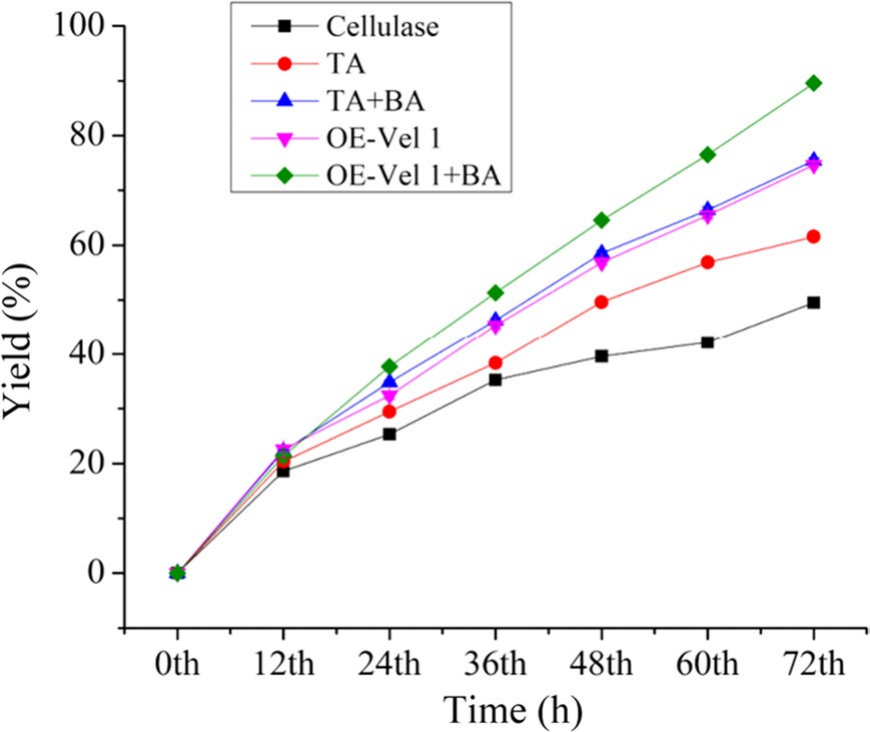
Hydrolysis of pretreated corn Stover by the crude enzyme of different approaches. Values are the average of biological triplicates. Error bars represent the standard error

The pretreated corn stover was hydrolyzed using crude enzymes of different samples (Fig. 4). The enzymes obtained from the TA *OE-Vel1* + BA showed maximum hydrolysis. This may be because of the high production of hydrolytic enzymes by the co-culture TA OE-Vel1 + BA. The over-expression of the *Vel1* gene in *T. asperellum* enriched the cellulase production. At 72 h, TA OE-*Vel1* + BA produced the hydrolysis yield of 89.56 ± 0.61%, which was greater than the co-culture of *T. asperellum* and *B. amyloliquefaciens* and the axenic culture of genetically engineered *T. asperellum.* The hydrolysis yield generated by the TA OE-*Vel1* + BA and TA + BA was higher than the axenic culture of *T. asperellum*. However, TA OE-*Vel1* + BA showed a better hydrolysis yield than TA + BA. This might be due to the reason of the activation of transcription factors and enzyme coding genes by the over expression of the *Vel1* gene and by the co-cultivation with *B. amyloliquefaciens* as an inducer. Consequently, the enzyme production and hydrolysis yield were higher in the TA OE-*Vel1* + BA sample.

### Synergistic effect of corn stover amendments and microbial inoculation on lignocellulose degradation, plant growth and defense response

Throughout pot experiment, maize plants were grown healthy without any toxic symptoms. The axenic and co-culture were used to enrich plant growth in soil with and without the amendments of corn stover (Additional file 1: Fig. S2). The growth parameters of plants grown in soil samples amended with corn stover differed significantly (P ≤ 0.05) from the plants grown in non-amended soil as assessed by Duncan’s new multiple range test. TA OE-*Vel1* + BA and TA + BA co-culture exhibited a remarkable effect on both plant growth and lignocellulolytic degradation. However, the plant growth in the untreated corn stover amended soil was reduced (Table 1). Overall, biodegradation of corn stover amended soil with TA OE-*Vel1* + BA co-culture (T13) increased the shoot height and root height of the maize plants when compared to non-amended soil and other treatments (Table 1). Shoot height and root height of maize plants grown in corn stover amended soil treated with co-culture (T12) were 1.68 and 1.31 fold higher, respectively than control (T1). likewise, the shoot height and root height of maize plants grown in non-amended soil treated with co-culture (T5) and TA OE-*Vel1* + BA co-culture (T6) were also higher than control (T1). Likewise, the fresh and dry biomass of shoot and root was also influenced by corn stover amendments treated with TA OE-*Vel1* + BA (T13) and TA + BA (T12). The influence of TA OE-*Vel1* + BA and TA + BA on the corn stover amendments improved the plant height and biomass of maize than all other treatment. The influence of TA OE-*Vel1* + BA and TA + BA co-culture on disease index against *Fusarium verticillioides* and *Cohilohorus herostrophus* was also observed in both amended and non-amended soil compared to the control (T7 and T14). On the other hand, the disease index of T12 and T13 were 7 times higher than that of control (T14).

**Table 1:** Effect of axenic, co-culture and modular co-culture of *T. asperellum* and *B. amyloliquefaciens* on the plant growth and biological control against *Fusarium verticillioides* and *Cohilohorus herostrophus* under both corn stover amended and non-amended soil in green house conditions

Treatments	Length (cm)		Wet weight (gm)		Dry weight (gm)		Disease index (%)	
	Shoot	Root	Shoot	Root	Shoot	Root	Root rot	Leaf spot
T1	41.22.41^i^	22.54 ± 0.36^f^	1.36 ± 0.04^f^	0.03 ± 0.07^f^	0.1 ± 0.02^g^	0.01 ± 0.021^e^	ND	ND
T2	54.26.3^f^	19.55 ± 0.6^h^	1.57 ± 0.09^f^	0.12 ± 0.08^d^	0.17 ± 0.01^f^	0.04 ± 0.06^b^	30^c^	30^c^
T3	51.98 ± 0.41^g^	31.17 ± 0.41^c^	2.51 ± 0.07^e^	0.14 ± 0.01^d^	0.33 ± 0.01^d^	0.0344 ± 0.01^c^	40^d^	40^d^
T4	55.46 ± 0.45^f^	25.8 ± 0.48^c^	3.26 ± 0.05^d^	0.12 ± 0.05^d^	0.33 ± 0.09^d^	0.04 ± 0.08^c^	30^c^	30^c^
T5	59.87 ± 0.5^d^	25.51 ± 0.2^e^	3.42 ± 0.07^d^	0.15 ± 0.01^d^	0.45 ± 0.07^c^	0.05 ± 0.01^b^	30^c^	30^c^
T6	65.55 ± 0.4^c^	28.99 ± 0.5^d^	4.4 ± 0.09^c^	0.15 ± 0.01^d^	0.46 ± 0.02^c^	0.05 ± 0.01^b^	20^b^	20^b^
T7	34.74 ± 0.20^j^	17.7 ± 0.29^ l^	2.5 ± 0.11^e^	0.04 ± 0.07e^f^	0.25 ± 0.01^e^	0.024 ± 0.05^d^	60^e^	60^e^
T8	43.94 ± 0.56^h^	21.02 ± 0.50^g^	2.72 ± 0.13^e^	0.08 ± 0.02^e^	0.28 ± 0.01^e^	0.02 ± 0.01^d^	ND	ND
T9	55.37 ± 0.37^f^	22.62 ± 0.47^f^	3.51 ± 0.15^d^	0.14 ± 0.015^d^	0.44 ± 0.01^c^	0.05 ± 0.01e^b^	30^d^	30^d^
T10	55.89 ± 0.37^e^	24.64 ± 0.51^e^	3.53 ± 0.14^d^	0.14 ± 0.06^d^	0.33 ± 0.17^d^	0.05 ± 0.01^b^	40^b^	40^b^
T11	68.88 ± 0.3^b^	35.55 ± 0.46^b^	5.68 ± 0.08^c^	0.22 ± 0.02^c^	0.44 ± 0.01^c^	0.05 ± 0.03^b^	20^a^	20^a^
T12	69.53 ± 0.36^b^	29.62 ± 0.51^d^	5.56 ± 0.15^b^	0.34 ± 0.15^b^	0.59 ± 0.02^b^	0.07 ± _0.016_^a^	10^a^	10^a^
T13	74.98 ± 0.57^a^	44.94 ± 0.28^a^	6.54 ± 0.14^a^	0.45 ± 0.017^a^	0.64 ± 0.011^a^	0.07 ± 0.0017^a^	10^a^	10^a^
T14	42.2 ± 0.3^j^	16.3 ± 0.42^J^	2.618 ± 0.1^e^	0.08 ± 0.004^e^	0.25 ± 0.001^e^	0.03 ± 0.001^c^	70^d^	70^d^

Results are average of five replicates for each treatment; the values given are the standard error of the mean. Different superscripts in the same column are significantly different (P < 0.05) based on the ANOVA

**Fig. 5:**
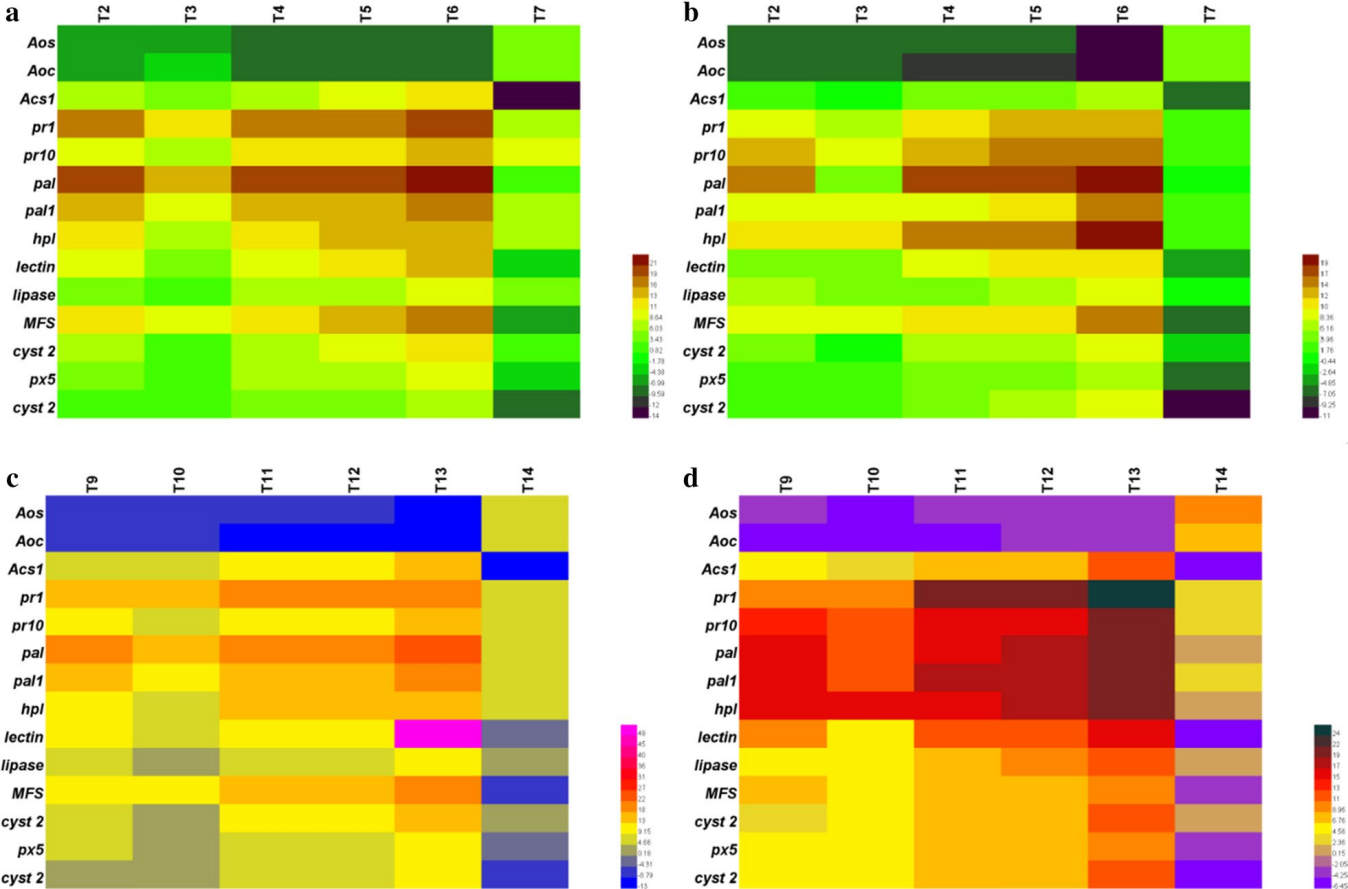
Induction of defense gene expression in maize roots and shoots by the axenic, co-culture and modular co-culture of *T. asperellum* and *B. amyloliquefaciens* against *Fusarium verticillioides* and *Cohilohorus herostrophus*. Heat map profile of defense gene expression in **a** root and **b** leaves of non-amended soil were showed in terms of fold changes compared to the control (T1). Heat map profile of defense gene expression in **c** root and **d** leaves of corn stover amended soil were showed in terms of fold changes compared to the control (T8). Results are average of five replicates for each treatment; the values given are the standard error of the mean

To further understand the plant response to the corn stover amended soil inoculated with co-culture, we studied the induction of defense-related gene expression using semi-quantitative reverse transcriptase (RT)-PCR (Fig. 5). The actin gene has been used as an internal control. Fourteen genes related to different plant defense pathways were selected: allene oxide synthase (AOS), allene oxide cyclase (AOC) (jasmonic acid), 1-aminocyclopropane-1-carboxylic acid synthase (ACS) (ethylene), pathogenesis-related protein 1 (PR1) and pathogenesis-related protein 10 (PR10) (systemic acquired resistance), phenylalanine ammonia-lyase (PAL) and (PAL1) (salicylic acid), hydroperoxide lyase (HPL), lectin, lipase, multiflux efflux synthase (MFS), cystatin ii proteinase inhibitor (Cyst2), peroxidase (PX5), cystatin proteinase inhibitor (Cyst) and thiolase (other defense-related genes). The regulation of these genes by axenic, TA OE-*Vel1* + BA and TA + BA co-culture were examined locally at the root and systematically at leaves. The defense gene expression against *Fusarium verticillioides* and *Cohilohorus herostrophus* on maize roots and leaves are shown in Fig. 5. The AOS and AOC gene was upregulated in both roots and leaves of plants infected with *Fusarium verticillioides* and *Cohilohorus herostrophus,* respectively, but it was gradually reduced in the plants treated with TA OE-*Vel1* + BA (T6 and T13), TA + BA (T5 and T12) co-culture, and axenic culture (T2, T3, T4, T9, T10 and T11) in both amended and non-amended soil. The upregulation of AOS and AOC revealed that the plants were highly infected by the *Fusarium verticillioides* and *Cohilohorus herostrophus* in T7 and T14. Based on the expression profiles, the ACS genes was highly induced by TA OE-*Vel1* + BA application in the root of T6 and T13 (Fig. 5a and c). Followed by the co-culture of TA + BA induced the ACS genes of maize plants. Interestingly, the expression of these genes was downregulated in T7 and T14. TA OE-*Vel1* + BA and TA + BA inoculated maize plants expressed the defense genes locally on the plant root and systematically in leaves as a response of *Cohilohorus herostrophus* (Fig. 5b and d). The expression of systemic acquired resistance pathway-related genes such as PR1 and PR10 in roots and leaves of maize plants inoculated with TA OE-*Vel1* + BA and TA + BA co-culture were upregulated than other treatments of both amended and non-amended soil. The PAL and PAL1 were upregulated in the following order T13 > T12 > T11 > T9 > T10 and T6 > T5 > T4 > T2 > T3 in both corn stover amended and non-amended soil, respectively. The upregulation of the genes such as HPL, lectin, lipase, MFS, Cyst2, PX5, Cyst, and thiolase was also enhanced by the TA OE-*Vel1* + BA and TA + BA co-culture compared to the control.

**Fig. 6:**
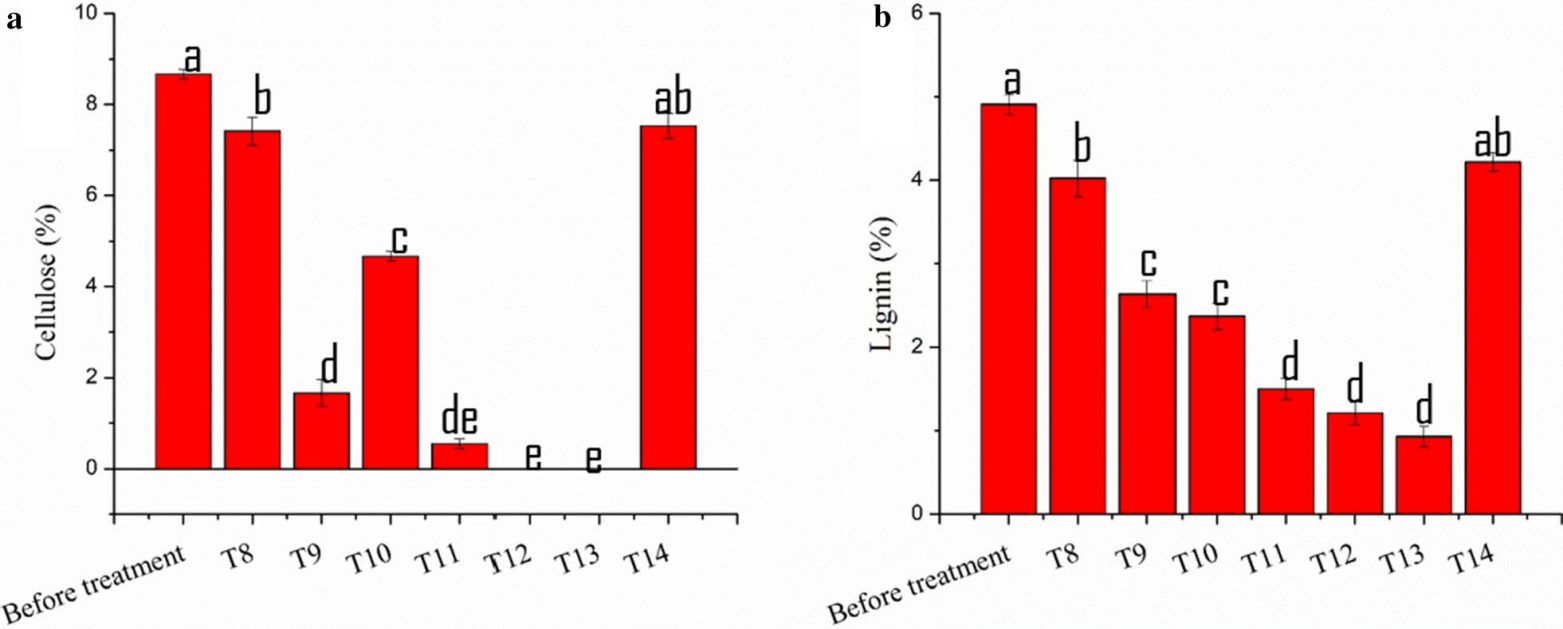
Effect of axenic, co-culture and modular co-culture of *T. asperellum* and *B. amyloliquefaciens* on the hydrolysis of cellulose (**a**) and lignin (**b**) content of corn stover amended soil. Results are average of five replicates for each treatment; the values given are the standard error of the mean. Different letters on the parentheses are significantly different (*P* ≤ 0.05)

**Fig. 7:**
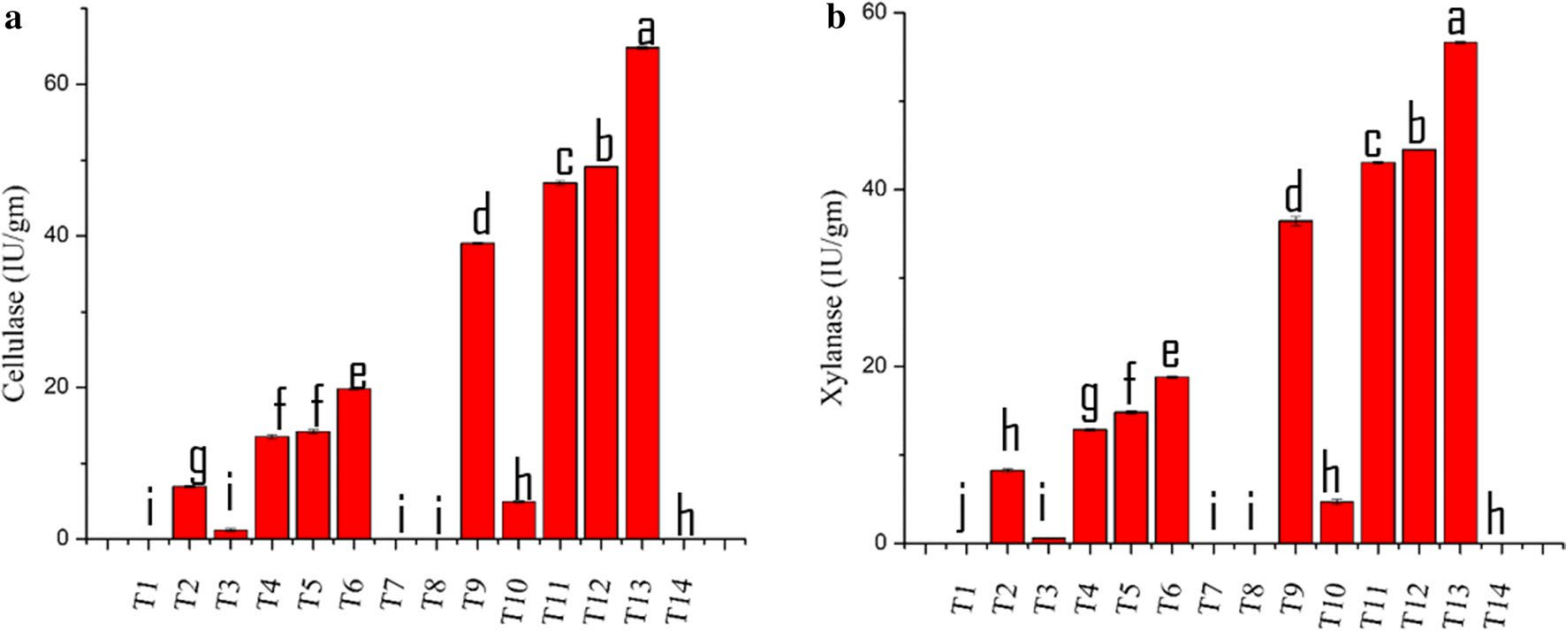
Effect of axenic, co-culture and modular co-culture of *T. asperellum* and *B. amyloliquefaciens* on the production of cellulase and xylanase of corn stover amended and non-amended soil. Results are average of five replicates for each treatment; the values given are the standard error of the mean. Different letters on the parentheses are significantly different (*P* ≤ 0.05)

SOM, TOC, TN and C/N content in the soil of each treatment are shown in Table 2. There were no notable changes in the content of soil SOM, TOC, TN and C/N in T1 to T7, which was not amended with the corn stover. Amendment of corn stover increased the soil SOM and TOC in the treatment T8, T14, and T10, which has not been treated with the *Trichoderma*. Biodegradation of corn stover amendment treated with TA OE-*Vel1* + BA and TA + BA co-culture reduced the SOM and TOC content of the soil. At the end of the experiments, the C/N ratio of the TA OE-*Vel1* + BA and TA + BA treated corn stover amendment soil was rapidly decreased and it was closer to the standard value compared soil treated with axenic culture and control. In comparison with all other treatments, T13 and T12 treatment showed better degradation. In connection to the improvement of the C/N ratio, the cellulose content of the T13 and T12 was completely reduced by the TA OE-*Vel1* + BA and TA + BA co-culture (Fig. 6a). Similarly, the lignin content was also reduced in T13 and T12 compared to other treatments and before treatment (Fig. 6b). The cellulase and xylanase content of the T13 and T12 was increased compared to other treatments (Fig. 7a and b).

**Table 2:** Effect of axenic, co-culture and modular co-culture of *T. asperellum* and *B. amyloliquefaciens* on the soil chemical properties such as SOM, TOC, TN and C/N ratio on both corn stover amended and non-amended soil

Treatments	SOM	TOC	TN	C/N
T1	358.11 ± 034^d^	716.23 ± 0.68^d^	31.25 ± 0.05^ab^	22.91 ± 0.02^e^
T2	348.65 ± 0.72^f^	697.3 ± 0.45^f^	31.24 ± 0.06^ab^	22.32 ± 0.02^f^
T3	353.03 ± 0.36^e^	706.07 ± 0.72^e^	31.16 ± 0.09^ab^	22.65 ± 0.04^ef^
T4	347.49 ± 0.19^f^	694.98 ± 0.38^f^	31.26 ± 0.03^ab^	22.22 ± 0.07^f^
T5	353.12 ± 0.26^e^	706.24 ± 0.52^e^	31.42 ± 0.04^a^	22.47 ± 0.02^ef^
T6	346.71 ± 0.26^f^	693.42 ± 0.52^f^	31.68 ± 0.15^a^	22.88 ± 0.09^fg^
T7	348.43 ± 0.32^f^	696.86 ± 0.65^f^	31.26 ± 0.04^ab^	22.28 ± 0.05^f^
T8	428.7 ± 0.21^a^	857.4 ± 0.43^a^	27.85 ± 0.4^f^	30.79 ± 0.4^a^
T9	347.66 ± 0.30^f^	695.33 ± 0.60^f^	29.26 ± 0.14^de^	23.76 ± 0.1^d^
T10	377.8 ± 0.35^c^	755.6 ± 0.7^c^	26.07 ± 0.19^ g^	28.98 ± 0.2^b^
T11	342.5 ± 0.6^g^	685 ± 0.30^g^	30.75 ± 0.28^bc^	22.27 ± 0.09^f^
T12	307.73 ± 0.25^i^	615.46 ± 0.51^i^	28.85 ± 0.28^e^	21.33 ± 0.2^g^
T13	326.78 ± 0.30^h^	653.56 ± 0.60^h^	30.32 ± 0.11^c^	21.55 ± 0.05^g^
T14	419.15 ± 0.31^b^	838.3 ± 0.62^b^	29.57 ± 0.29^d^	28.35 ± 0.2^c^

Results are average of five replicates for each treatment; the values given are the standard error of the mean. Different superscripts in the same column are significantly different (P < 0.05) based on the ANOVA

## Discussion

The establishment of genetic engineering technology has allowed the production of an extensive array of bio-products by exploiting several microbes as biocatalysts [21]. Even though considerable application of bacterial or fungus cultures has been well studied, genetic engineering technology has satisfied the growing need for complex biosynthesis enzymes. On the other hand, co-cultivation of microorganism has been effectively used to convert the complex substrates into the simple biomolecules for the industrial applications [22–25]. In recent times, the genetically engineered microbial strains are used in co-cultivation to enhance the specific metabolites through metabolic pathways [11]. In connection with the point previously mentioned, integration of co-cultivation and genetic engineering compromises the biosynthesis of enzymes required for the hydrolysis of agricultural biomass onsite to improve sustainable agriculture. This new approach provides a space to develop new engineered biosynthetic pathways in co-cultivation environment [26].

In the present study, we investigated the role of *T. asperellum Vel1* gene on production of the carbohydrate active enzymes, including FPAase, CMCase, PNPCase, PNPGase, xylanase I and xylanase II. The Carbohydrate Active Enzyme (CAZYmes) refers to the enzymes, which are required to hydrolyze the polysaccharides. *Vel1* is a comprehensive regulator of the numerous fungi, especially T*richoderma*. Interestingly, *Vel1* is involved in the regulation of asexual sporulation, secondary metabolism, and mycoparasitism of *Trichoderma* [29]. Karimi Aghcheh et al., [8] confirmed that the expression of cellulase requires *Vel1* gene. Similarly, we observed that the overexpression of the *Vel1* gene increased the production of cellulase. Our results further showed that the cellulases and xylanases are co-regulated by the *Vel1*. The enzyme activity of the different approaches revealed that it coincides with the expression of genes such as cellulase and xylanase encoding genes and transcription regulating factors. The expression pattern of *cbh1*, *cbh2*, *egl1*, *egl2*, *bgl1*, *xyn1* and *xyn2* was analyzed to know the regulatory level of cellobiohydrolases, endoglucanases, β-glucosidase and xylanase. The results assumed that overexpression of the *Vel1* gene positively influenced the expression of these genes to increase the activity of cellobiohydrolases, endoglucanases, β-glucosidase, and xylanase.

The regulation of cellulase and hemicellulase gene expression of the *Trichoderma* is extremely synchronized by the transcription regulatory factors [20]. In *Trichoderma*, ten transcription factors were identified as important for regulation of cellulase gene expression [20]. Among them, *XYR1*, *ACE II* and *ACE III* are significant transcriptional regulators. [15–17, 27]. Also, the *HAP2/3/5* complex stimulates the open chromatin structure required for the transcription stimulation [20]. *BglR* stimulates the β-glycosidases genes. Interestingly, in our study, these genes were upregulated in co-ordination with the *Vel1* gene. *ACE1* and *RCE1* are the transcriptional repressor of cellulase gene expression [19, 28]. The carbon catabolic repression has been regulated by the negative regulation of *CRE1* gene [18]. Transcript data of *ACEI*, *RCE1*, and *CRE1* of the present study were downregulated in the TA OE-*Vel1* strain (Fig. 3). Similarly, the co-cultivation (TA + BA and TA OE-*Vel1* + BA) downregulated the expression of *ACEI*, *RCE1*, and *CRE1*. The decreased transcription of *ACEI*, *RCE1*, and *CRE1* by co-cultivation could positively upregulated expression of *cbh1*, *cbh2*, *egl1*, *egl2*, and *bgl1*. Overall, the present investigation demonstrated that the regulatory action of the *Vel1* on the production of CAZymes. The additional role of the *Vel1* gene concerning the synthesis of cellulolytic enzymes is fascinating due to the interaction of several transcription regulatory genes.

However, interaction between the *Vel1* gene, CAZymes and transcription regulating factors is not acquainted so far. Conversely, the interactions between sporulation and cellulase production have been proven in *T. reesei* [8]. In this connection, it is exciting that several cellulase related genes and its transcription regulatory genes are clustered in the genome of *T. asperellum*. It had proved that these genes were positively regulating cellulase and xylanase production. Further, the results proved that the *Vel1* gene is a superior regulatory gene to synchronize the expression of cellulases and other related transcription factors [9, 29]. Moreover, we observed that the co-cultivation is also positively influencing this synchronization.

The co-cultivation with *B. amyloliquefaciens* offered differentiated cellular environs to induce the genes involved in metabolic pathways [12, 13]. The metabolic pathway might comprise several enzymes, and these properties could vary depending on the circumstance. Axenic cultivation offers an identical environment and it might not be appropriate to express all genes. Similar to our study, the co-cultivation of an engineered *Escherichia coli* and *Saccharomyces cerevisiae* also enhanced cellulase production [30]. In addition to that, modular co-culture engineering decreases the intrusion of biosynthesis of other metabolites and induce the specific genes. In the present study, *B. amyloliquefaciens* induced the gene expression of cellulase and xylanase encoding genes.

The enzymes synthesized by the different samples generated more glucose than the commercial cellulase. It showed that on-site enzyme production has several advantages including cost-effective, CAZymes composition and concentration [31]. Further, it improved the hydrolysis efficiency of pre-treated corn stover. Consequently, the samples applied for cellulase synthesis and hydrolysis of pre-treated corn stover were compared and shown in Additional file 1: Table S2. The enzymes produced by TA OE-*Vel1* + BA co-culture (sample 4) were confirmed to be efficient and cost-effective to produce glucose from the corn stover. The co-cultivation of genetically engineered *T. asperellum* and *B. amyloliquefaciens* is a promising method to hydrolyse the lignocellulolytic biomass for agricultural purposes. Finally, the co-culture of TA OE-*Vel1* + BA increased the consumption of complex substrates and enhanced the hydrolysis rate of pretreated corn stover. Furthermore, the co-culture of the genetically engineered *T. asperellum* and *B. amyloliquefaciens* have a better impact on the colonization of agro residues due to the multiple functions along with the maximum production of hydrolysis enzymes. Hence this has been used to recycle the crop residues in pot based experiments for the betterment of soil fertility, plant growth and disease resistance.

Retention of biomass residue after harvest is an important module of sustainable agriculture practice. Presently, in China the practice of maize retention has been followed to improve the soil properties and yield of the subsequent crop [32]. The accumulation of crop residues lacking appropriate soil management could lead to problems including temporary loss of nitrogen and moisture. Hence, new technology for the retention of crop residue is required to improve the growth of a subsequent crop. The application of microbes into the soil after retaining the maize residues showed the benefits on the soil quality and subsequent plant growth. The application of *Streptomyces microflavus* and *Aspergillus niger* enriched the degradation of lignocellulose biomass and stimulates soil nutrient availability for the subsequent plant growth [32, 33]. Hence, 25% of maize residue was incorporated into the soil and treated with the axenic, TA OE-*Vel1* + BA and TA + BA co-culture to enrich the biomass degradation for subsequent plant growth, defense potential and disease control. TA OE-*Vel1* + BA and TA + BA co-culture increased the residue decomposition through the production of lignocellulolytic enzymes and thereby it increased the nutrients content of the soil.

In general, the incorporation of maize residues increases the soil C/N ratio and reduce the availability of nitrogen to plants [34]. Hence, some researchers suggested to incorporate the inorganic nitrogen into the soil to maintain the optimal C/N for plant growth [34]. In the present study the TA OE-*Vel1* + BA and TA + BA co-culture reduced the C/N ratio closer to the standard value compared to the axenic culture without any supplementation of inorganic nitrogen. Besides, few studies showed that the retention of maize residues in the field increased the wheat growth, grain filling and yield compared to the non-retention field [32, 35]. Similarly, the plant growth parameters are higher in the corn stover amended soil compared to the non-amended soils. The improvement in enzyme production by the TA OE-*Vel1* + BA and TA + BA co-culture significantly correlated with the soil quality and plant growth (Additional file 1: Table S3). The *Vel1* overexpression gene upregulated the expression of plant defense gene. Mukherjee and Kenerley (2010) observed that the knockout of *Vel1* gene did not mycoparasite the pathogens *R. solani* and *P. ultimum* due to the downregulation of secondary metabolite producing genes. The present investigations revealed that the overexpression of the *Vel1* gene might induce the secondary metabolite producing genes of *Trichoderma* and mycoparasitism to upregulate the defense potential of plants. In addition, the *B. amyloliquefaciens* present in the co- culture is an another plant growth promoting and biocontrol agent. This *B. amyloliquefaciens* not only induced the production of *Trichoderma* enzymes and corn stover degradation, but it was also involved in the plant growth and biocontrol efficiency when it was applied in the soil. Previously, Karuppiah et al. [12] evidenced that the co-culture stimulated the defense potential of plants against pathogens. Likewise, we observed that the TA OE-*Vel1* + BA and TA + BA improved the plant defense gene expression than the axenic cultures. Overall, the *in-vivo* study revealed that the amendment of corn stover with the TA OE-*Vel1* + BA and TA + BA co-culture of *T. asperellum* and *B. amyloliquefaciens* into soil increased the production of soil enzymes and cornstover degradation. The increment of soil organic carbon and maintenance of the C/N ratio increased the soil fertility and maize growth than the non-amended soil. The application of these plant growth and biocontrol microbes in the form of co-culture not only increased the plant growth but also enhancing the expression of defense genes against *Fusarium verticillioides* and *Cohilohorus herostrophus* pathogens and reduced the disease index compared to other treatments. This residue management technology could be useful to retain the agricultural biomass after harvesting to preserve the soil structure, and productivity and it can be an option of eco-friendly technology for the effective utilization of plant residue.

## Conclusions

The *Vel1* gene expression is increased with the help of the TrpC promotor. The enzyme activity such as FPAase, CMCase, PNPCase, PNPGase, xylanase I and xylanase II were increased by the over expression of *Vel1.* It also increased the hydrolysis of pretreated corn stover (1.8 fold). The present study confirmed that the regulation of cellulase and xylanase was coordinated by the regulation of several transcription factors (Fig. 3). Further, it was also confirmed that *Vel1* gene co-ordinate the regulation of the transcription factor to induce the cellulase and xylanase encoding genes for the maximum production of enzymes. The co-cultivation of genetically modified *T. asperellum* and *B. amyloliquefaciens* increased (2.1 fold) the production of cellulase and xylanase to hydrolyse the cellulolytic biomass. Our results revealed that the treatment of TA OE-*Vel1* + BA on the corn stover amended soil increased the soil lignocellulolytic enzyme activity and corn stover degradation. The TA OE-*Vel1* + BA positively influenced the growth of maize plants and disease resistance against *Fusarium verticillioides* and *Cohilohorus herostrophus*. The co-cultivation of genetically engineered *T. asperellum* and *B. amyloliquefaciens* could be used as a novel and advanced technique to return the crop residue into the field to improve the soil fertility along with the plant growth and disease resistance. This technique could be an eco-friendly technology for efficient consumption of crop residue and plant growth in the field for the next level of sustainable agriculture.

## Methods

### Microbial strains

The *T. asperellum* GDFS1009 (CGMCC NO. 9512) and *B. amyloliquefaciens* 1841 (CGMCC NO. 15465) were acquired from our culture collection facility and Sichuan University, respectively and stored in the CGMCC, China. *T. asperellum* GDFS1009 and *B. amyloliquefaciens* were cultured on potato dextrose (PD) and luria bertani (LB) agar, respectively. *Agrobacterium tumefaciens* AGL-1 has been used for the agrobacterium mediated transformation of pCAMBIA1300 *Vel1* OE vector into *T. asperellum*. *Fusarium verticillioides* and *Cohilohorus heterostrophus* were sourced from our laboratory microbial collection center and used as a target pathogen to induce the root rot and leaf spot diseases in maize plants.

### Engineering of *Vel1* gene overexpression

A 2.17 kb of DNA portion comprising the *TrpC* promoter, and *TrpC* terminator from the pCAMBIA1300 vector and *Vel1* ORF (pCAMBIA1300) of *T. asperellum* GDFS1009 was over-lapped using PCR. This gene cassette was introduced into pCAMBIA1300 to generate the pCAMBIA1300 *Vel1* OE via Hieff CloneTM One Step Cloning Kit. The *T. asperellum Vel1*OE strains were attained by transforming the pCAMBIA1300 *Vel1*OE into *T. asperellum* GDFS1009. The mutants were confirmed by PCR. Primers used for the engineering of *Vel1* gene are shown in Additional file 1: Table S4.

### Cellulase production by the mono and co-culture

For the mono-culture, *T. asperellum* (TA) (10^6^/mL spores) or the recombinant *T. asperellum* (TA OE-*Vel1*) (10^6^/mL spores) were cultured in the minimal broth containing 2% avicel as described by [36]. For co-cultivation, 0.1% of the *B. amyloliquefaciens* 1841 (BA) was added into the 48^th^ hour *T. asperellum* and recombinant *T. asperellum* pre-culture medium and named as TA + BA and TA OE-*Vel1* + BA respectively. Further, it was cultured in the incubator shaker at 30^◦^C until 72h.

### Enzyme activity

Filter paper activity (FPAase) was analyzed based on the standard method explained by Ghose [37]. The endoglucanase (CMCase) activity was tested according to Bailey and Nevalainen [38]. Cellobiohydrolase (pNPCase), and β-glucosidase (pNPGase) were assessed by the methodology of Zhang et al. [36]. Xylanase I and II were assessed at pH 3.7 and 5.0 respectively according to the method of Sticker et al. [39]. All testing was executed in biological triplicates.

### Gene expression analysis

RNA isolation and cDNA synthesis were carried out as described by Karuppiah et al. [11]. The expression of cellulose and hemicellulose hydrolysis related genes and transcription regulators were estimated as described by Karuppiah et al. [11]. The real-time data were normalized with the 18S rRNA gene by means of the 2^−ΔΔCt^ method. All tests were carried out in three independent experiments in triplicate. The list of primers used has given appears in Additional file 1: Table S5.

### Pretreatment and hydrolysis of corn stover

The corn stover was pretreated by the method of Tsegaye et al. [40]. The crude enzymes of four different samples were used to hydrolyze the lignocellulose biomassas suggested by Zhang et al. [36]. The glucose content of the hydrolysate was estimated using HPLC (Waters 410, Waters, MA) and eluted with 0.004 M H_2_SO_4_ at a flow rate of 0.6 mL/min.

## Synergistic effect of corn stover amendments and microbial inoculation on lignocellulose degradation, plant growth and defense response

### Experimental setup

The pot trial was conducted in a greenhouse in 15 cm diameter pots comprising of horticulture soil amended with or without 25 mg corn stover g^−1^ soil (25%). The corn stover was air-dried, finely powdered, and uniformly mixed into the soil. The pot trial was comprised of 14 different experiments with triplicates. The treatments were: T1- control, T2-TA, T3-BA, T4- TA OE-*Vel1*, T5-co-culture of TA and BA, T6- co-culture of TA OE-*Vel1* and BA, T7- pathogen, T8- 25% corn stover amendment (control), T9- 25% corn stover amendment + TA, T10- 25% corn stover amendment + BA, T11- 25% corn stover amendment + TA OE-*Vel1*, T12- 25% corn stover amendment + co-culture of TA and BA, T13- 25% corn stover amendment + co-culture of TA OE-*Vel1*and BA, T14- 25% corn stover amendment + pathogen. The axenic and co-cultures were evenly applied in 25% corn stover amended and non-amended soil. All experimental pots were watered everyday untill 15 days. After that, maize seeds were seeded and grow until 30 days, 1 × 10^6^ conidia ml^−1^ of *Fusarium verticillioides* and *Cohilohorus heterostrophus* was applied into the pot soil and leaves, respectively on T2—T7 and T9—T14. The plant growth and disease index were evaluated according to the method of Karuppiah et al. [11] and Wang et al. [41]. The disease index was accessed based on the leaf spot and root rot disease of each treatment using the grading technique from grade 0 to grade 5. 0: no disease; 1: no more than 10%; 2: 11–30%; 3: 31–50%; 4: 51–70%; and 5: > 70%. the disease index was calculated as follows: DI = Σ (sum of plants in each disease stage × grade value)/sum of all plants × uppermost grade × 100) [41].

Expression of defense genes by the axenic and co-culture in both corn stover amended and non-amended soils against the pathogens were analyzed using qPCR. Roots and leaves of each treated maize plants were individually collected and the RNA was extracted with the Vazyme fastpure plant total RNA isolation kit. The cDNA was synthesized using Vazyme HiScript III 1st Strand cDNA Synthesis Kit (+ gDNA wiper). The amplification was performed as described previously by [13] using Roche light cycler 96. The *Actin* gene was used to normalize the gene expression. Data are expressed using the 2^−ΔΔCT^ method.

Determination of soil organic matter, total organic carbon, lignin, cellulose, and C/N ratio.

Soil organic matter (SOM) was estimated gravimetrically by the loss‐on‐ignition technique and expressed as mg ^−g^ dry weight as suggested by Danise et al. [42]. Total organic carbon (TOC) was calculated using the results of SOM with 2 as a conversion factor from SOC to TOC [42]. Total nitrogen (TN) was estimated using the Kjeldahl method [43]. C:N ratio was calculated using the results TOC and TN. Lignin and cellulose content of the soil was estimated using the Updegra and acetyl‐bromide spectrophotometric technique, respectively as suggested by Danise et al. [42].

### Determination of cellulase and xylanase activity in soil

Humic materials of the soil samples were removed using active carbon and PolychIal AT according to the method of Kanazawa and Miyashita [44]. Soil cellulase activity was determined with the modification of Kanazawa and Miyashita [44] method. Briefly, 10 gm of soil and 200 mg of avicel were dissolved with phosphate buffer in a conical flask and incubated for 24 h at 28 °C. After incubation, the samples were centrifuged and reducing sugar content of the 0.5 m1 of the supernatants was estimated using 3 ml of DNS reagent. To determine the soil xylanse activity, 10 gm of soil and 200 mg of xylan were dissolved with phosphate buffer in a conical flask and incubated for 24 h at 28 °C. After incubation, the samples were centrifuged, and reducing sugar content was measured as described above [45]. One unit of cellulase and xylanse activity is explained as the quantity of enzyme requisite to liberate 1 µmol reducing sugars per gram of soil.

### Statistical analysis

The graphs were plotted using origin 6.0. Results displayed were mean of triplicate values through standard error. The Student’s T-test was conducted to differentiate the gene expression among the control and test samples. Two-way ANOVA, post hoc LSD, and Duncan test were used to determine the statistical significance between each samples through SPSS 2.0. Pearson’s correlation was performed between the variables of all treatments used in pot experiments using SPSS 2.0.

Statistical significances were determined using one-way analysis of variance (ANOVA) and post hoc Duncan multiple range tests (DMRTs).

## Supplementary Information

**Additional file 1**:** Table S1**. The growth and cellulase production of Trichoderma asperellum recombinants on CBH screening medium.** Table S2**. Comprehensive information about the enzyme activity and corn stover hydrolysis of different samples.** Table S3**. Correlation plot of Pearson correlation coefficient for all measured variables in pot experiments. ** Correlation is significant at the 0.01 level (2-tailed). *Correlation is significant at the 0.05 level (2-tailed).** Table S4**. Primers used in construction of over-expression strains.** Table S5**. Sequences of the primers used for the real-time PCR.** Figure S1**. Work flow of the present study.** Figure S2**. Table 1 Effect of axenic, co-culture and modular co-culture of T. asperellum and B. amyloliquefaciens on the plant growth and biological control against Fusarium verticillioides and Cohilohorus herostrophus under both corn stover amended and non-amended soil in green house conditions. (T8-T14) cornstover amended soil; (T1-T7) cornstover non amended soil.

## Data Availability

All data generated during this study are included in this published article.

## Abbreviations

ACS: 1-Aminocyclopropane-1-carboxylic acid synthase
AOC: Allene oxide cyclase
AOS: Allene oxide synthase
BA: *Bacillus amyloliquefaciens*
*bgl1*: β-Glucosidase
C/N: Cabon/Nitrogen
*cbh1*: Cellobiohydrolases 1
*cbh2*: Cellobiohydrolases 2
CMCase: Endoglucanase
Cyst: Cystatin proteinase inhibitor
Cyst2: Cystatin ii proteinase inhibitor
*egl1*: Endoglucanases 1
*egl2*: Endoglucanases 2
FPAase: Filter paper activity
HPL: Hydroperoxide lyase
MFS: Multiflux efflux synthase
PAL1: Phenylalanine ammonia-lyase (PAL)
PNPCase: Cellobiohydrolase
PNPGase: β-Glucosidase
PR1: Pathogenesis-related protein 1
PR10: Pathogenesis-related protein 10
PX5: Peroxidase
SOM: Soil organic matter
TA: *Trichoderma asperellum*
TA OE-*Vel1*: Overexpression mutant of *Trichoderma asperellum Vel1* locus
TA OE-*Vel1* + BA: Co-culture of TA OE-*Vel1* and BA
TA + BA: Co-culture of *Trichoderma asperellum* and *Bacillus amyloliquefaciens*
TN: Total nitrogen
TOC: Total organic carbon
*Vel1*: Velvet 1
*xyn1*: Xylanase 1
*xyn2*: Xylanase 2
