# Supplementary material for: Co-culture of Vel1-overexpressed Trichoderma asperellum and Bacillus amyloliquefaciens: An eco-friendly strategy to hydrolyze the lignocellulose biomass in soil to enrich the soil fertility, plant growth and disease resistance
Source: Microb Cell Fact. 2021 Mar 2;20:57. doi: 10.1186/s12934-021-01540-3 (PMC7927390; doi:10.1186/s12934-021-01540-3)
Supplement: Supplementary file 1 — Additional file 1: Table S1. The growth and cellulase production of Trichoderma asperellum recombinants on CBH screening medium. Table S2. Comprehensive information about the enzyme activity and corn stover hydrolysis of different samples. Table S3. Correlation plot of Pearson correlation coefficient for all measured variables in pot experiments. ** Correlation is significant at the 0.01 level (2-tailed). *Correlation is significant at the 0.05 level (2-tailed). Table S4. Primers used in construction of over-expression strains. Table S5. Sequences of the primers used for the real-time PCR. Figure S1. Work flow of the present study. Figure S2. Table 1 Effect of axenic, co-culture and modular co-culture of T. asperellum and B. amyloliquefaciens on the plant growth and biological control against Fusarium verticillioides and Cohilohorus herostrophus under both corn stover amended and non-amended soil in green house conditions. (T8-T14) cornstover amended soil; (T1-T7) cornstover non amended soil. [file 12934_2021_1540_MOESM1_ESM.docx]

**Supplementary Table 1. The growth and cellulase production of *Trichoderma asperellum* recombinants on CBH screening medium**

| **Transformants** | **Growth and cellulase production on cellulase screening medium** | | | |
| --- | --- | --- | --- | --- |
| **Colony diameter (mm)^a^** | 0-3 | 3-5 | 5-7 | 8-9 |
| **Number of transformants** | 5 | 49 | 67 | 5 |
| **FPA (FPIU/mL)^b^** | <5 | 5-7.5 | 7.5-10 | 10-13.5 |

a medium was prepared with microcrystalline cellulose. Colony growth was measured in millimeter after 5 days; Values are the average of biological triplicates.

b FPA (FPIU/mL) was measured after 6th day of fermentation in the minimal medium containing 2% avicel. Values are the average of biological triplicates

**Supplementary Table 2. Comprehensive information about the enzyme activity and corn stover hydrolysis of diﬀerent samples.**

| Method | 1 | 2 | 3 | 4 |
| --- | --- | --- | --- | --- |
| FPA (FPIU/mL) | 6.25 | 7.92 | 12.45 | 15.91 |
| CMCase (IU/mL) | 40.15 | 54.16 | 62.14 | 73.04 |
| PNPCase (IU/mL) | 2.98 | 3.24 | 4.95 | 6.32 |
| PNPGase (IU/mL) | 1.99 | 2.56 | 3.26 | 4.45 |
| Xylanase I (IU/mL) | 52.56 | 63.23 | 73.67 | 83.56 |
| Xylanase II (IU/mL) | 53.87 | 61.57 | 67.78 | 78.45 |
| Hydrolysis Yield (%) | 61.56 | 75.45 | 74.63 | 89.56 |
| Glucose (g/L) | 36.38 | 44.6 | 44.11 | 52.94 |

**Supplementary Table 3.** **Correlation plot of Pearson correlation coefficient for all measured variables in pot experiments. ** Correlation is significant at the 0.01 level (2-tailed). * Correlation is significant at the 0.05 level (2-tailed).**

|  | | Cellulase | Xylanase | SOM | TN | TOC | CN | Shoot length | Root length | Shoot wet weight | Root wet weight | Shoot dry weight | Root dry weight | Disease index |
| --- | --- | --- | --- | --- | --- | --- | --- | --- | --- | --- | --- | --- | --- | --- |
| Cellulase | Pearson Correlation | 1 | .998^**^ | -.640^**^ | -.008 | -.640^**^ | -.466^**^ | .840^**^ | .766^**^ | .884^**^ | .868^**^ | .855^**^ | .763^**^ | -.427^**^ |
|  | Sig. (2-tailed) |  | .000 | .000 | .960 | .000 | .002 | .000 | .000 | .000 | .000 | .000 | .000 | .005 |
|  | N | 42 | 42 | 42 | 42 | 42 | 42 | 42 | 42 | 42 | 42 | 42 | 42 | 42 |
| Xylanase | Pearson Correlation | .998^**^ | 1 | -.648^**^ | .001 | -.648^**^ | -.476^**^ | .846^**^ | .749^**^ | .879^**^ | .857^**^ | .851^**^ | .771^**^ | -.430^**^ |
|  | Sig. (2-tailed) | .000 |  | .000 | .995 | .000 | .001 | .000 | .000 | .000 | .000 | .000 | .000 | .004 |
|  | N | 42 | 42 | 42 | 42 | 42 | 42 | 42 | 42 | 42 | 42 | 42 | 42 | 42 |
| SOM | Pearson Correlation | -.640^**^ | -.648^**^ | 1 | -.400^**^ | 1.000^**^ | .903^**^ | -.612^**^ | -.574^**^ | -.489^**^ | -.608^**^ | -.534^**^ | -.534^**^ | .421^**^ |
|  | Sig. (2-tailed) | .000 | .000 |  | .009 | .000 | .000 | .000 | .000 | .001 | .000 | .000 | .000 | .005 |
|  | N | 42 | 42 | 42 | 42 | 42 | 42 | 42 | 42 | 42 | 42 | 42 | 42 | 42 |
| TN | Pearson Correlation | -.008 | .001 | -.400^**^ | 1 | -.400^**^ | -.753^**^ | .018 | .134 | -.121 | -.096 | -.106 | -.187 | .040 |
|  | Sig. (2-tailed) | .960 | .995 | .009 |  | .009 | .000 | .911 | .399 | .444 | .546 | .502 | .235 | .802 |
|  | N | 42 | 42 | 42 | 42 | 42 | 42 | 42 | 42 | 42 | 42 | 42 | 42 | 42 |
| TOC | Pearson Correlation | -.640^**^ | -.648^**^ | 1.000^**^ | -.400^**^ | 1 | .903^**^ | -.612^**^ | -.574^**^ | -.489^**^ | -.608^**^ | -.534^**^ | -.534^**^ | .421^**^ |
|  | Sig. (2-tailed) | .000 | .000 | .000 | .009 |  | .000 | .000 | .000 | .001 | .000 | .000 | .000 | .005 |
|  | N | 42 | 42 | 42 | 42 | 42 | 42 | 42 | 42 | 42 | 42 | 42 | 42 | 42 |
| CN | Pearson Correlation | -.466^**^ | -.476^**^ | .903^**^ | -.753^**^ | .903^**^ | 1 | -.446^**^ | -.470^**^ | -.296 | -.399^**^ | -.340^*^ | -.299 | .268 |
|  | Sig. (2-tailed) | .002 | .001 | .000 | .000 | .000 |  | .003 | .002 | .057 | .009 | .028 | .055 | .086 |
|  | N | 42 | 42 | 42 | 42 | 42 | 42 | 42 | 42 | 42 | 42 | 42 | 42 | 42 |
| Shoot length | Pearson Correlation | .840^**^ | .846^**^ | -.612^**^ | .018 | -.612^**^ | -.446^**^ | 1 | .819^**^ | .855^**^ | .851^**^ | .833^**^ | .857^**^ | -.561^**^ |
|  | Sig. (2-tailed) | .000 | .000 | .000 | .911 | .000 | .003 |  | .000 | .000 | .000 | .000 | .000 | .000 |
|  | N | 42 | 42 | 42 | 42 | 42 | 42 | 42 | 42 | 42 | 42 | 42 | 42 | 42 |
| Root length | Pearson Correlation | .766^**^ | .749^**^ | -.574^**^ | .134 | -.574^**^ | -.470^**^ | .819^**^ | 1 | .788^**^ | .792^**^ | .737^**^ | .583^**^ | -.562^**^ |
|  | Sig. (2-tailed) | .000 | .000 | .000 | .399 | .000 | .002 | .000 |  | .000 | .000 | .000 | .000 | .000 |
|  | N | 42 | 42 | 42 | 42 | 42 | 42 | 42 | 42 | 42 | 42 | 42 | 42 | 42 |
| Shoot wet weight | Pearson Correlation | .884^**^ | .879^**^ | -.489^**^ | -.121 | -.489^**^ | -.296 | .855^**^ | .788^**^ | 1 | .827^**^ | .919^**^ | .784^**^ | -.390^*^ |
|  | Sig. (2-tailed) | .000 | .000 | .001 | .444 | .001 | .057 | .000 | .000 |  | .000 | .000 | .000 | .011 |
|  | N | 42 | 42 | 42 | 42 | 42 | 42 | 42 | 42 | 42 | 42 | 42 | 42 | 42 |
| Root wet weight | Pearson Correlation | .868^**^ | .857^**^ | -.608^**^ | -.096 | -.608^**^ | -.399^**^ | .851^**^ | .792^**^ | .827^**^ | 1 | .853^**^ | .843^**^ | -.398^**^ |
|  | Sig. (2-tailed) | .000 | .000 | .000 | .546 | .000 | .009 | .000 | .000 | .000 |  | .000 | .000 | .009 |
|  | N | 42 | 42 | 42 | 42 | 42 | 42 | 42 | 42 | 42 | 42 | 42 | 42 | 42 |
| Shoot dry weight | Pearson Correlation | .855^**^ | .851^**^ | -.534^**^ | -.106 | -.534^**^ | -.340^*^ | .833^**^ | .737^**^ | .919^**^ | .853^**^ | 1 | .851^**^ | -.309^*^ |
|  | Sig. (2-tailed) | .000 | .000 | .000 | .502 | .000 | .028 | .000 | .000 | .000 | .000 |  | .000 | .047 |
|  | N | 42 | 42 | 42 | 42 | 42 | 42 | 42 | 42 | 42 | 42 | 42 | 42 | 42 |
| Root dry weight | Pearson Correlation | .763^**^ | .771^**^ | -.534^**^ | -.187 | -.534^**^ | -.299 | .857^**^ | .583^**^ | .784^**^ | .843^**^ | .851^**^ | 1 | -.317^*^ |
|  | Sig. (2-tailed) | .000 | .000 | .000 | .235 | .000 | .055 | .000 | .000 | .000 | .000 | .000 |  | .041 |
|  | N | 42 | 42 | 42 | 42 | 42 | 42 | 42 | 42 | 42 | 42 | 42 | 42 | 42 |
| Disease index | Pearson Correlation | -.427^**^ | -.430^**^ | .421^**^ | .040 | .421^**^ | .268 | -.561^**^ | -.562^**^ | -.390^*^ | -.398^**^ | -.309^*^ | -.317^*^ | 1 |
|  | Sig. (2-tailed) | .005 | .004 | .005 | .802 | .005 | .086 | .000 | .000 | .011 | .009 | .047 | .041 |  |
|  | N | 42 | 42 | 42 | 42 | 42 | 42 | 42 | 42 | 42 | 42 | 42 | 42 | 42 |

**Supplementary Table 4. Primers used in construction of over-expression strains**

| **Primers** | **sequence（5'-3')** |
| --- | --- |
| **pro--F** | TAAAACGACGGCCAGTGCCAGGAGGTCAACACATCAAT |
| **pro-R** | TGGACGGCGTCGCCATTTGGATGCTTGGGTAGAA |
| **ove-vel1-F** | TTCTACCCAAGCATCCAAATGGCGACGCCGTCCA |
| **ove-vel1-R** | CACATTATTATGGAGAAATAGCCCATTCATGTTGCCCTC |
| **term-F** | GAGGGCAACATGAATGGGCTATTTCTCCATAATAATGTG |
| **term-R** | GACCTCCGAATTCGAGCTCGAAATTGACGCTTAGAC |

**Supplementary Table 5. Sequences of the primers used for the real-time PCR.**

| **Gene** | **Forward (5’-3’)** | **Reverse (5’-3’)** | **Reference** |
| --- | --- | --- | --- |
| **ace3** | GTATGTATTATCGGAGTTGCTGAG | CTCGCCATCGTCGTCAATAG | (Zhang et al., 2019) |
| **hap2** | TAYGTNAAYGCNAARCART | CKCATRGCGTGGTTGTG | (Zeilinger et al., 2001) |
| **hap3** | GARCARGAYMGNTGGYTNCCNAT | TCNGCRTARTTYCRAANCC |  |
| **hap5** | TAYACNGGNACNTGGGCNAAYGT | ATRTCDATNAGRAARTCRAACAT |  |
| **lae1** | ACTGGAGATTGACTGGATGC | TTCTGCGTCTGGTAGCCTC | (Karimi-Aghcheh et al., 2013) |
| **vel1** | AGCCTTATGTGCCTCACT | GCAGGAGCAGAGTAGTTG |  |
| **bg1r** | TGGATAGCTACTACCAGGGC | CAGACTAAGGGGACGTTGT | (Nitta et al., 2012) |
| **rce1** | CCGTCTTCAAGCAGTACCGT | GAGACCACAGGTTGGACTGG | This study |
| **ace1** | GGACGAGGAGGAGATTATG | GTGAGTCTTCTCGTGCTT | (Portnoy et al., 2011) |
| **ace2** | GACAAGAAGCTCAGGTGTC | ACTGTGTTCATGGCTGTG |  |
| **xyr1** | **CTTCCTCCTCCTGCTCATCG** | **TCGTGTGCCCTAACAATGGTC** | (Zhang et al., 2018) |
| **cre1** | GTCACCGTCATCAAGCCCAA | AGGGCACTTGTAGGGACGAG |  |
| ***cbh1*** | CTCCATCTCCGAGGCTCTTACC | GCAAGTGCCGCCATATCTGTTAT |  |
| ***cbh2*** | GCATATTACGCCTCTGAAGTTAGCA | GCATAGTTACCGCCATTCTTGTTG |  |
| ***egl1*** | AACTACCGCTGGATGCACGA | TAGTCGACGCCCTCGATGAA |  |
| ***egl2*** | TGAACAAGTCCGTGGCTCCAT | ACAATTCGTAGGTCCGCTCCAA |  |
| ***bgl1*** | CAAGTGACTGGTGCCGAGGTA | CGTTGCTGTTCCGCTCTGAC |  |
| ***xyn1*** | GGTTGGACGACTGGATCT | GGTTGTCCTCCATGATGTAG |  |
| ***xyn2*** | CATCGTCGAGAACTTTGGCA | GCGTGCGGTAAATGTCGTAG |  |
| ***swol*** | CCAAACTATACGAGTAGCC | GAGTGAATGTCTTGATGG |  |
| ***18S rRNA*** | GGTGGAGTGATTTGTCTG | CTTACTAGGGATTCCTCG | (Tisch et al., 2011) |


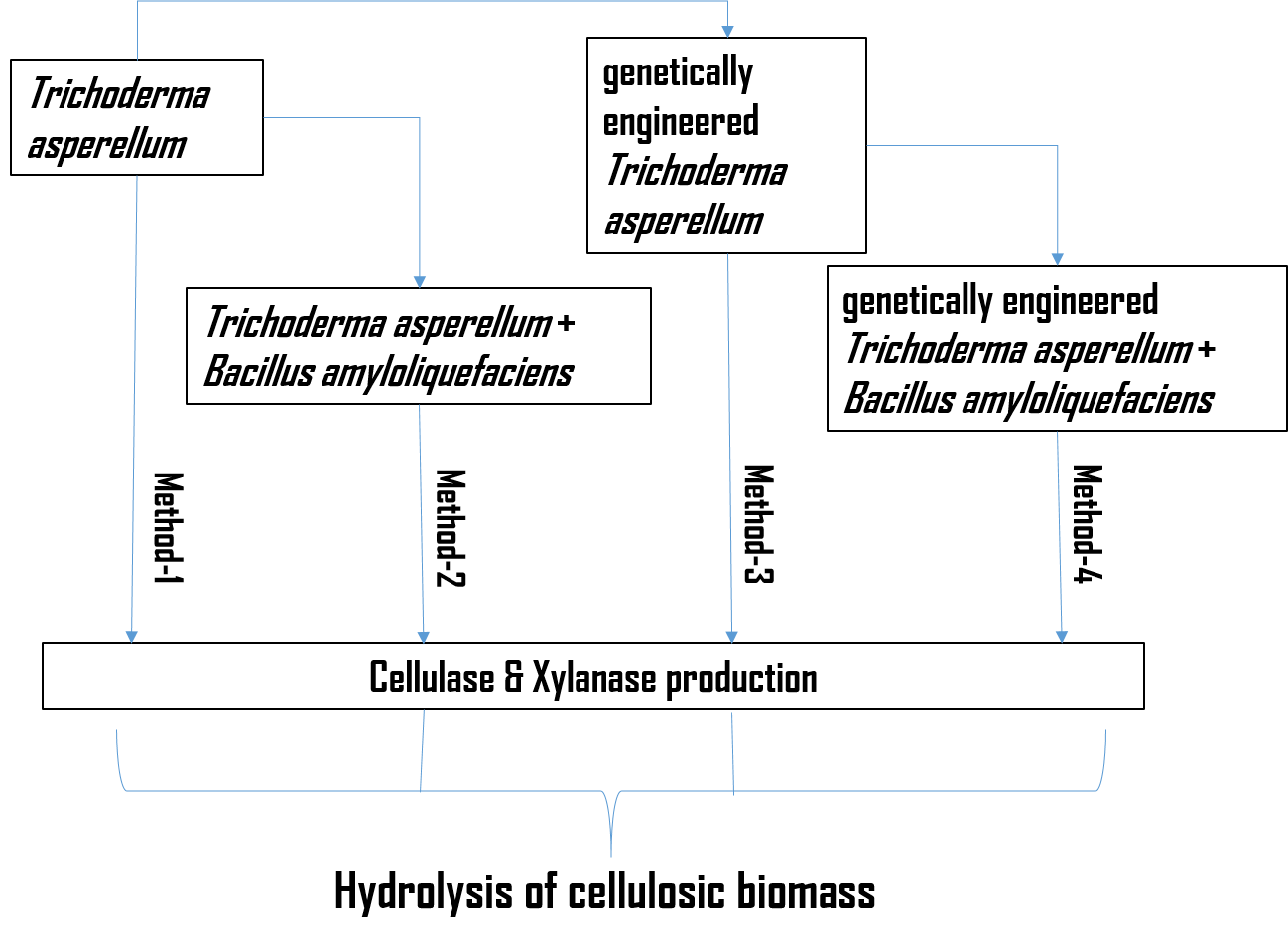


**Supplementary figure 1. Work flow of the present study**

**
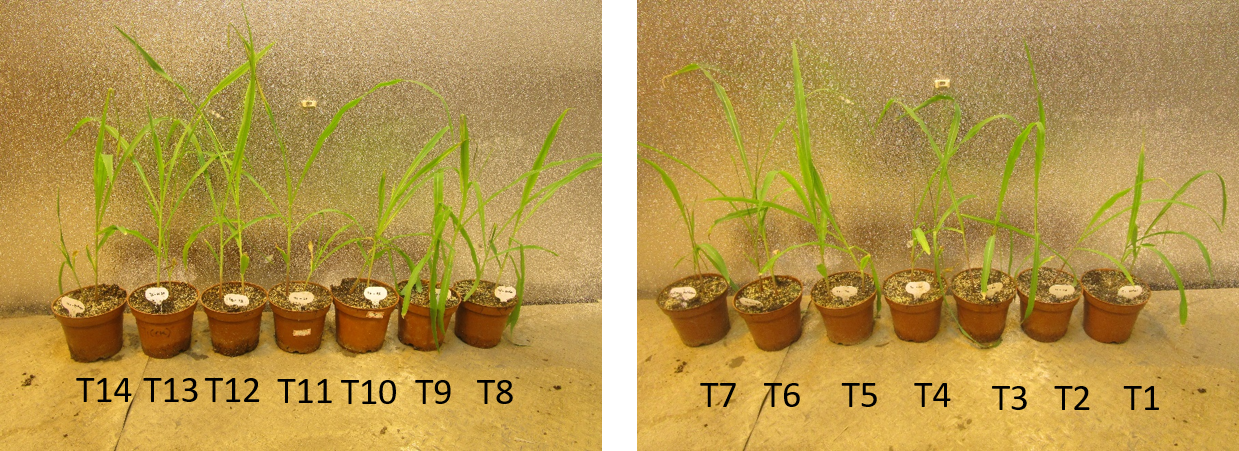
**

**Supplementary figure 2. Table 1 Effect of axenic, co-culture and modular co-culture of T. asperellum and B. amyloliquefaciens on the plant growth and biological control against *Fusarium verticillioides* and *Cohilohorus herostrophus* under both corn stover amended and non-amended soil in green house conditions. (T8-T14) cornstover amended soil; (T1-T7) cornstover non amended soil**

**References**

Karimi-Aghcheh, R., Bok, J.W., Phatale, P.A., Smith, K.M., Baker, S.E., Lichius, A., Omann, M., Zeilinger, S., Seiboth, B., Rhee, C., Keller, N.P., Freitag, M., and Kubicek, C.P. (2013). Functional analyses of Trichoderma reesei LAE1 reveal conserved and contrasting roles of this regulator. *G3 (Bethesda)* 3**,** 369-378.

Nitta, M., Furukawa, T., Shida, Y., Mori, K., Kuhara, S., Morikawa, Y., and Ogasawara, W. (2012). A new Zn(II)2Cys6-type transcription factor BglR regulates β-glucosidase expression in Trichoderma reesei. *Fungal Genetics and Biology* 49**,** 388-397.

Portnoy, T., Margeot, A., Seidl-Seiboth, V., Le Crom, S., Chaabane, F., Linke, R., Seiboth, B., and Kubicek, C. (2011). Differential Regulation of the Cellulase Transcription Factors XYR1, ACE2, and ACE1 in Trichoderma reesei Strains Producing High and Low Levels of Cellulase. *Eukaryotic cell* 10**,** 262-271.

Tisch, D., Kubicek, C.P., and Schmoll, M. (2011). New insights into the mechanism of light modulated signaling by heterotrimeric G-proteins: ENVOY acts on gna1 and gna3 and adjusts cAMP levels in Trichoderma reesei (Hypocrea jecorina). *Fungal Genetics and Biology* 48**,** 631-640.

Zeilinger, S., A, E., T, M., Mach, R., and Kubicek, C. (2001). The Hypocrea jecorina HAP 2/3/5 protein complex binds to the inverted CCAAT-box (ATTGG) within the cbh2 (cellobiohydrolase II-gene) activating element. *Molecular genetics and genomics : MGG* 266**,** 56-63.

Zhang, J., Chen, Y., Wu, C., Liu, P., Wang, W., and Wei, D. (2019). The transcription factor ACE3 controls cellulase activities and lactose metabolism via two additional regulators in the fungus Trichoderma reesei. *Journal of Biological Chemistry*.

Zhang, J., Zhang, G., Wang, W., Wang, W., and Wei, D. (2018). Enhanced cellulase production in Trichoderma reesei RUT C30 via constitution of minimal transcriptional activators. *Microbial Cell Factories* 17**,** 75.
